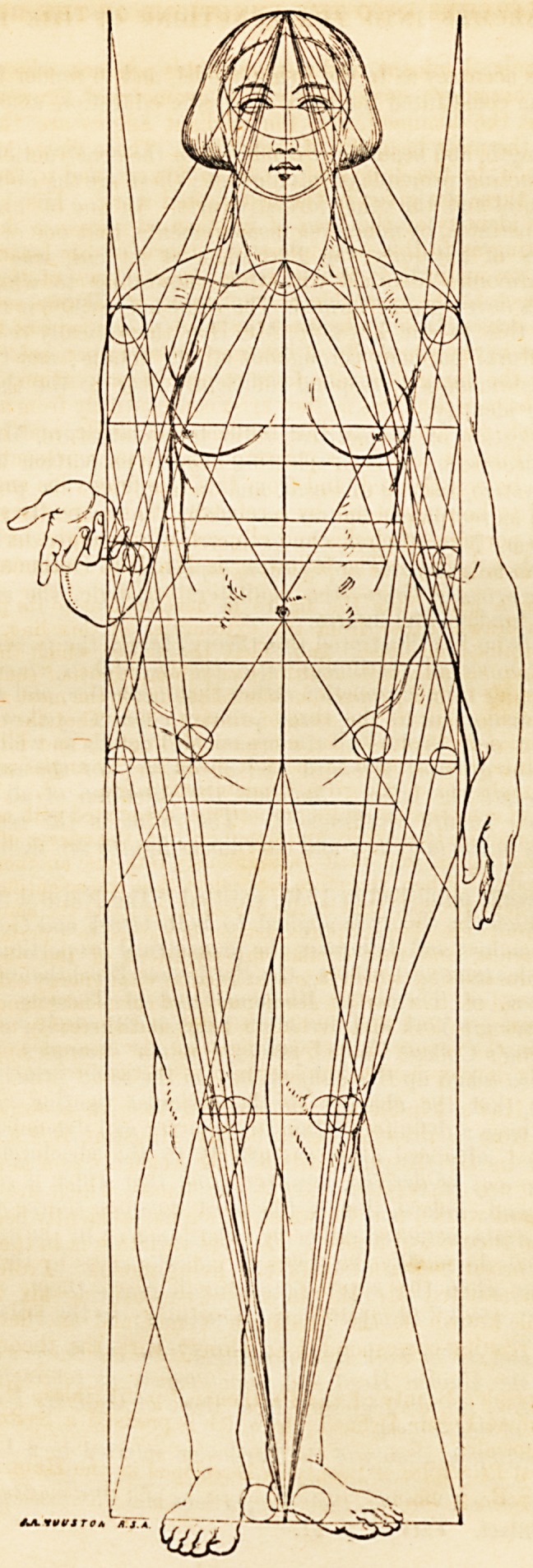# Researches into the Functions of the Brain

**Published:** 1855-10-01

**Authors:** Thomas Laycock

**Affiliations:** Physician to the York Dispensary, &c.


### RESEARCHES INTO THE FUNCTIONS OF THE BRAIN.

BY THOMAS LAYCOCK, M.D.,

*Physician to the York Dispensary, &c.*

We are permitted by the kindness of Mr. Churchill to reprint from the last number of the "British and Foreign Medico-Chirurgical Review," with the sanction of its distinguished and accomplished writer, the subjoined valuable and important paper on the "Functions of the Brain." We are anxious to bring Dr. Laycock's highly ingenious and original observations before the readers of the "Psychological Journal," who naturally take a special and deep interest in this department of cerebral physiology. This essay is highly suggestive. We need not observe that if Dr. Laycock is able fully to substantiate his position, a new light will be thrown upon this department of Physiological Science.—[Ed.]

It is now some years since I extended to the cerebrum the doctrines current as to the reflex function of the spinal cord. During the interval which has elapsed, cerebral physiology has sufficiently advanced to warrant an attempt at extending my views into the more metaphysical and obscure regions of consciousness and thought. By way of retrospect, I may be permitted to observe that when engaged in an investigation of certain morbid conditions of the nervous system, as they were presented to my notice nearly twenty years ago in the wards of the York County Hospital (of which I was for some time the resident medical officer), the imperfect nature of the views then current as to cerebral physiology, and their inadequacy to explain or elucidate functional diseases of the brain, were continually forced upon me. Physiology afforded hardly any clue to the pathology of mental derangement in any of its forms of reverie and somnambulism, whether natural or artificial, or of those varied morbid manifestations of the consciousness, the perceptions, and the will, which are grouped under the terms hysteria, mesmeric phenomena, &c. Mental philosophy and metaphysics were even less instructive than physiology, for the sum of the practical knowledge they imparted, as to the function of the brain in mental operations, might be stated in the words of Reid: "In perception, the object produces some change in the organ [of special sense]; the organ produces some change upon the nerve; and the nerve produces some change in the brain." The nature of that change, and its relations to the consciousness and the will, appeared to be wholly unknown to mental philosophers, and were only discussed when it was sought to establish some vague and profitless hypothesis. Nay, not a few metaphysicians hardly concede so much as the fundamental proposition, that the brain is the organ of mind, and necessary to the manifestation of its phenomena; for they practically ignore the science of cerebral physiology, and investigate the operations of mind as if the brain took no part in them. How dangerous to scientific and religious truth and morals such a fundamental error may be, is in process of demonstration by the proceedings of "spiritualists" and their congeners, who deduce the wildest and most mischievous doctrines from their experimental researches.

Feeling this want of definite knowledge as to the functions of the brain, and its relation to mental phenomena, when investigating cerebral pathology, I endeavoured to attain to something better, by adopting the inductive method of inquiry. Facts and experimental researches in abundance were not wanting; and I therefore soon reached this general conclusion, that the brain being a congeries of ganglia, did not differ in its laws of action from the other ganglia of the nervous system; and in particular, that like the spinal ganglia, it was subject to the laws of *reflex* action. It followed, therefore, that although, as the organ of conscious mind, its functions were carried on *with* consciousness, yet as being a series of ganglia analogous to the spinal, its functions might be, and often were, carried on *without* consciousness, or at least independently of the will, and of the accompanying sensations, if consciousness existed. This doctrine having been, in the main points, approved and adopted by eminent physiologists and pathologists (amongst whom my friend, Dr. Carpenter, holds a very high rank), may be considered as established; for, although I still stand almost alone in maintaining that in the so-called *sensational* actions, sensation or consciousness takes no share causally, and is only a coincident phenomenon not necessary to the acts, the main proposition, that cerebral action goes on unconsciously, is placed on an irrefragable basis. I would particularly refer to Dr. Carpenter's very lucid demonstration of this part of the doctrine in the fourth edition of his "Principles of Human Physiology," §§ 805—845, and his admirable applications of it to various forms of cerebral disorder, whether arising spontaneously or induced artificially.

On one point, however, I am obliged to differ from Dr. Carpenter—namely, that there is an "*essential* distinction, both in their anatomical and physiological relations, between the sensory ganglia and the cerebrum, or hemispheric ganglia." It has been, on the contrary, a fixed and fundamental doctrine with me, that as to reflex action, there is no *essential* distinction of the kind, the differences being, anatomically and physiologically, rather that of species than genus; nay, that there is no essential distinction in the mode of action of all organized structures, whether animal or vegetable, considered in relation to the *fundamental psychological phenomenon* of reflex action, *the intelligent respondence to stimuli.* So that the laws of reflex action apply to every form of organism, however lowly, and whether it be a plant or an animal; to every kind of tissue, however simple, and whether it be merely a congeries of cells, or be so highly developed and endowed as the vesicular neurine of the human hemispherical ganglia. Indeed, I need only repeat here what I have previously stated.

"The doctrine of a molecular organization within organized structures, such as that it shall correspond and be appropriate to given stimuli received by appropriate organs, necessarily constitutes the basis of all inquiries into the laws of action in those structures. And there can be no doubt, such is the magnificent uniformity in the infinite diversity of creation, that the laws of action of the agent and reagent in vital phenomena are as definite as those operating on chemical phenomena, could we but effect a sufficiently minute analysis and induction."*

It is only, in short, on the deductions from this all-comprehensive generalization that the basis for a practical and sufficient system of human psychology can be laid.† It may be stated, then, as an admissible general proposition, and therefore of universal experience, that the cerebrum (the organ of thought) may be put into the same modes of action as occur in the other ganglia of the nervous system, when they are rendered active, independently of the will or the consciousness, by their appropriate stimuli; and (to use Dr. Carpenter's words) may act upon impressions transmitted to it, and convey elaborate results, such as we might have attained by the purposive (or volitional) direction of our minds to the subject, *without any consciousness* on our own parts; so that we only become aware of the operation which has taken place, when we compare the result, as it presents itself to our minds after it has been attained, with the materials submitted to the process. To those who have carefully observed the phenomena of thought in relation to the will and the consciousness, this mode of mental action must be a familiar fact; and to those who have studied the phenomena of reflex action, especially as displayed in the instincts of animals, its dependence upon the cerebral functions must be perfectly obvious and comprehensible. On the one hand, therefore, we have consciousness; on the other, unconscious yet intelligent action. These are the *psychological* phenomena. As the common medium of both, we have the cerebrum, the functions of which, in relation to these phenomena, form, therefore, the *physiological* problems to be investigated.

As a preliminary step, some statement of what is meant by reflex phenomena and of their nature is necessary. It has long been known, that animals so mutilated as to be deprived of consciousness, and even mere segments of animals, display, when irritants are applied to the integument, or to the special apparatuses, movements of as definite a character as those which are directed by the will, or are under the guidance of sensations. Very numerous experimental vivisections have been made from time to time, to determine the

* Appendix to Essay on Reflex Function of the Brain, § 3. "British and Foreign Medical Review," vol. xix. p. 308.

† This doctrine has been stated by me on different occasions. In an article on Hysteria (the last of a series), published in the "Edinburgh Medical and Surgical Journal," No. 140, July, 1839, I advocated the identity of function of all vital structures, whether vegetable or animal, ganglionic or cerebral. Again, in my "Treatise on the Nervous Diseases of Women," 1840, (to illustrate which I first commenced these researches), chapters vi.—viii. inclusive are devoted to the elucidation of this doctrine; chap viii. being headed, "The instinctive actions in relation to consciousness—the brain subject to the laws of reflex action." At the meeting of the British Association, in York, in 1844, I read the paper on the "Reflex Function of the Brain" above mentioned; and in the correspondence with Mr. George Combe, which arose out of the views advanced therein, I again reiterated the doctrine, extending it to reflex nutrition and development. "The development, conservation, and reproduction of all organism," I show, "are regulated by an unerring law of design—a law as generally applicable to living matter as the law of gravity to universal matter."—"Lancet," vol. ii. 1845, p. 256.

true nature of these movements, especially on cold-blooded vertebrata, in which class of vertebrates they are the most obvious. Whytt was one of the earliest of modern physiologists to institute these experiments. He found that "a frog lives and moves its members for half an hour after its head is cut off; nay, when the body of a frog is divided in two, both the anterior and posterior extremities preserve life and a power of motion for a considerable time." Whytt found, also, that although the brain was not necessary to these movements—for they may be continually excited in headless frogs—they were no longer manifested if the spinal cord was destroyed. Whytt observed similar adapted movements in vipers, and believed that they were necessarily connected with *sensation*.

"We have no other way," says he, "to satisfy ourselves that an animal is alive or endued with feeling, than by observing whether it shows uneasiness when anything hurts or tends to destroy any of its parts, and an endeavour to remove or avoid it. Since, therefore, the bodies of vipers make just the same kind of motions, when pricked with a sharp instrument, two or three days after losing their head, heart, and other bowels, as if they were entire, we are naturally led to conclude that they are still in some sense alive, and endowed with feeling—i. e., animated by a sentient principle."

This deduction from the phenomena was adopted by the majority of physiologists after Whytt—as Haller, Cuvier, Dumas, Alison, Le Gallois—and was, in fact, the doctrine generally current until Dr. Hall renewed attention to the subject, and made these experimental vivisections the basis of an improved pathology of certain diseases of the nervous system, specially implicating the *motor* system. He argued *that they were wholly independent of sensation*, and successfully; for there are few modern physiologists who agree with Whytt, Haller, and the rest. There was a contemporary of Haller, however, who gave a most lucid and complete exposition of the whole doctrine of the reflex action of the nervous system, carrying it far beyond the views of Dr. Hall, and extending it to the whole phenomena of animal life. This was J. A. Unzer, whose "Erste Gründe" is still the best work of reference on the subject, and still unapproached by modern physiologists.* Prochaska's "Commentaries" are but a free summary of Unzer's views, with the more metaphysical and really the more important portion omitted. It was Unzer who first systematically showed the identity of mere reflex phenomena with those that are instinctive and emotional, and explained the share which the states of the consciousness, termed pleasure and pain, have in all these excited acts. He also, of all neurologists, has most successfully made these doctrines elucidate the highest mental phenomena.

The fundamental principles of motor reflex action are these:—That there is an apparatus so contrived as to place the individual in relation with the external world, and receive impressions from it in such a way that, whatever in the external world is good for the organism, is sought after and secured, if possible; and whatever is injurious is avoided or repelled, if possible; secured or repelled automatically and mechanically, without the intervention of any sensation, feeling, thought, volition, or act of conscious mind whatever. That the adapting and *quasi*-rational or sentient agent which combines and regulates the movements of the limbs or other organs to these ends is seated, in nervated animals, in the masses of nerve-cells (vesicular neurine) termed ganglia. That the apparatus by which it acts, consists: *a*. Of a special histological arrangement and constitution of the vesicular neurine in each ganglion, in virtue of which it responds to stimuli according to a fixed and predetermined plan; *b*. Of a special histological arrangement and constitution of the vesicular neurine

* I had the honour and pleasure to translate and edit this work, together with Prochaska's "Commentaries," for the Sydenham Society.

on the periphery of the organism, which, coming into contact with the external world, is influenced according to a predetermined plan, and transmits the changes thus induced to the ganglionic vesicular neurine along conductors—the afferent nerves; *c*. Of efferent nerves (distributed to distant organs) which commence within the ganglionic vesicular neurine, and by the changes within which they, in their turn, are influenced, according to a fixed and predetermined plan, transmitting these influences to the motor system; *d*. Of the muscular system, which, receiving through the efferent nerves the influences originating in the ganglionic vesicular neurine, contracts in part, or as a whole, and in so doing puts in motion the varied mechanism already constructed, so that the external world is acted upon through the latter, intelligently and adaptively, to a distinct purpose and object—the preservation in well-being of the individual of the species. The primary object, therefore, of the reflex function of the nervous system is, psychologically, "*nostri conservatio*," to use the expressive phrase of Prochaska; the essentially necessary means of its attainment is automatic histological action within masses of vesicular neurine, according to a definite arrangement, and a fixed and predetermined series of changes.

We might rest here, and be content with stating that the cause (or necessary antecedent) to the infinitely varied and exquisitely adapted actions and movements known as reflex, automatic, unconscious, and instinctive, is this definite arrangement and fixed mode of action of the vesicular neurine; but the mind at once perceives the incompleteness of the statement, for it is obvious that there must be a necessary antecedent to the intelligent *action* of the machinery, in the intelligent *construction* of it. If we watch ever so superficially the growth and development of organisms, we are struck by the never-ceasing and ever-varied manifestation of the highest order of intelligence, from the first formation of the primordial cell to the perfect evolution of the entire mechanism of the individual. It is unnecessary to recapitulate illustrations of this general fact. The phenomena it includes have been the source of every variety of speculative philosophy, from Plato downwards; they are the basis of all natural theology; they are the great facts of geology, zoology, and natural history; and are ever connected, in all speculations, with the *instincts*—that is, the intelligent but unconscious *use* of the instruments thus intelligently but (to the individual) unconsciously *constructed*. With the hypotheses and speculations of metaphysical theology and speculative philosophy the inductive method has no sort of connexion—it is the great *fact* that alone concerns us, that there is inherent in the primordial cell of every organism, whether it be animal or vegetable, and in all the tissues which are developed out of it, an intelligent power or agent, which acting in all cases independently of the consciousness of the organism, and whether the latter be endowed with consciousness or not, forms matter into machines and machinery of the most singular complexity with the most exquisite skill and of wondrous beauty, for a fixed, manifest, and predetermined object—namely, the preservation and welfare of the individual, and the continuance of the species. This *quasi*-intelligent agent thus works with an apparently perfect knowledge of number, geometry, mathematics, and of the properties of matter as known to the human intellect under the term "natural philosophy" or physics—that is to say, with a perfect knowledge of chemistry, electricity, magnetism, mechanics, hydraulics, optics, acoustics—but as far transcending the limited knowledge of the human intellect, as the structures and adaptations of living organisms exceed in beauty and fitness the most finished works of man. Speculation apart, and the fact alone considered that such mental powers, so unconsciously acting, are inherent in every form of organized matter, it need no longer be considered novel or surprising that the unconscious operations of the human cerebrum attain to the perfection they sometimes do attain, or that the blind instincts of animals are so complete, and display so much knowledge of the external world.

The relation between the machines of organisms thus constructed and their actual uses, manifested in reflex phenomena, is too immediate and direct to doubt that the construction and use depend alike upon the same cause. In further developing my views, I shall have occasion to bring forward ample proofs and illustrations of this proposition, but I may here state that, if we were to divide the two classes of phenomena, and assign different causes to each, as has been the custom hitherto, we should only wander away into the hypotheses of speculative philosophy and metaphysical theology, leaving behind us the firm ground of fact and induction, and excluding ourselves from the large and perfectly untrodden field of research which the doctrine advanced opens out to us. I therefore take it as an established principle, that the *quasi*-intelligent agent which operates in the construction of organisms directs the use of the organs constructed.

Having thus traced the intelligent construction and use of organs in living organisms to an unconsciously acting principle of intelligence, as the common source of both, and having identified the results of the unconscious use (or reflex phenomena) with the results of that form of *cerebral* action which is carried on unconsciously,—or, in other words, having shown that the latter are reflex in their nature, it follows, necessarily and obviously, that these reflex *cerebral* phenomena are dependent upon the operation of the same unconsciously acting agent which constructs organs—or, in other words, the unconsciously acting mind of the cerebrum, and the intelligent agent from which constructive and reflex phenomena originate, are identical in their nature and operation. This proposition is the logical and inevitable deduction from the premises; I may add, that it is the logical and inevitable induction from facts, as I shall shortly proceed to demonstrate.*

We have, then, an unconsciously acting principle of intelligence operating upon or through matter in three modes. 1. It moulds and compounds matter into living organisms according to a fixed, predetermined, and unchanging sequence of phenomena or plan, having for its object the good of the individual or of the species, forming machines to this end of great complexity and wonderful adaptability out of simple material elements, and arranging the living

* In thus using the terms "unconscious," and "unconsciously acting," I mean them solely to indicate the mental state of the organism itself. An unconsciously acting principle of intelligence is not a new idea, paradoxical as it may appear, for so the soul itself has been designated by modern psychologists. Thus Morell, "The soul, as we have shown, is *prior* to consciousness. It exists *unconsciously* from the formation of the first cell-germ; it operates *unconsciously* throughout all the early processes of life; it acts *unconsciously* even in the greater part of the efforts which subserve our intellectual development."—"Elements of Psychology," p. 75. Again, —"The same principle which shows itself in the human organization—which gives form and feature to the body—which adapts all the organs to their several purposes —which constructs the nervous system as the great medium of mental manifestation—which implants the instincts and prompts the senses to their appropriate work—this principle rises in due time to a *self-conscious* activity, in which it can recognise its own Divine origin, and aspire towards its own equally Divine destination."—Ibid. p. 77. Consciousness is, in fact, but one form of *manifestation* of the principle of intelligence. I know of no one word which will *exactly* designate the latter; I, therefore, shall merely use that phrase, or that of unconscious agent. With this strict limitation I may even be permitted to use the phrase unconscious *mind*, synonymously with the phrase unconscious principle of intelligence; mind being, when thus used, synonymous with the "soul" of psychologists. The great source of misapprehension, as Morell remarks, is the notion which confounds the human soul with the human *consciousness*.

structures in such a way that these machines act with the greatest precision and fitness to the purpose for which they are constructed. 2. It *moves* and *regulates* these machines according to fixed, predetermined, and unchanging sequences of phenomena, one change necessarily exciting another by sequential association according to a pre-arranged plan, having for its object the good of the individual or of the species. 3. In animals endowed with consciousness, it acts upon the vesicular neurine contained within the cranium, which it has already constructed, according to a fixed and predetermined order of change, one change necessarily exciting another by sequential association; the *results* of which changes, or series of changes, are presented to the consciousness, and constitute, in part, at least, the phenomena of thought. This is a summary of the actual operations of the unconsciously acting principle of intelligence, irrespective of all theory.

The next step in this inquiry is, to determine the relations which mind and its operations bear to the unconscious principle and its operations. For this purpose, the threefold division just given will be our best guide, for the operations of the mind may be classed also under three corresponding heads—viz., 1. It designedly seeks to subdue and mould matter to its requirements, using for its designings those mental powers or faculties generalized under the term *intellectual*, and which have a *knowledge* of cause and effect, or of the necessary order of events, as the basis of all their operations. 2. It regulates, by an act of will, the current of its thoughts, and the movements of its own bodily organs in their operation (whether mediately or immediately) on the external world. 3. In these processes of thought and of will it acts upon or through the vesicular neurine contained within the cranium, controlling by its means the action of the muscles, and through it attaining to self-consciousness and knowledge of the external world. The problem to solve is, what are the relations, or rather the phenomena, manifested in common by the two forms of intelligence?

First, as to the unconsciously *constructing* principle and its operations. Its phenomena may be considered from a twofold point of view—*i.e.*, as they are manifested in the body itself, in relation with consciousness simply; or abstractedly, as the results of an intelligent agent, and in relation, therefore, with the intellectual powers or faculties of the mind. In regard to the influence of the *constructive* principle of organisms upon the consciousness, little is known, and, as to the majority, little can be known; for with regard to them, it is not possible to say whether consciousness exists or not. Construction, in the sense I use the term, is not limited to *development*, or the first formation of organs, but properly includes *nutrition* (which, strictly speaking, is a continual reconstruction) and separation. The state of the consciousness in *development*, so far as it is manifested in the developing organism, is clearly a state of pleasure. We know nothing of its existence in embryonic or intra-uterine life; but during the period of growth (in all mammals, at least) the operations of the unconsciously constructing principle are associated with physical enjoyment, or a pleasurable feeling of existence. The same condition is observed, but perhaps in a less intense degree, during the process of continued reconstruction, so long as the objects and intentions of the constructing principle are attained. Should, however, its predetermined plans be interrupted, by an imperfect constitution or supply of the nutrient materials, the general feeling of physical *well*-being is changed into one of *ill*-being. At the same time, special painful feelings are felt, in correlation with the efforts of the constructive principle, to obviate the interruption to its predetermined plans; and the sensations of hunger, thirst, want of air, of exercise, of repose, &c., are induced. With these are associated acts and efforts to attain the means by which the predetermined arrangements (which are those of the healthy state) may again come into operation, constituting the *instinctive acts*, or the so-called reflex phenomena, when directed to the external world; and the operations of the so-called VIS MEDICATRIX NATURÆ, when directed to the working of the inner system of machinery. It is not possible to separate these two classes of conservative phenomena, except in this way—*i.e.*, as to the sphere of action of the unconsciously acting principle of intelligence; in respect to their object and origin, they are identical. The effort to supply fluid to the blood (the instinct of thirst), when it is wanted to carry off by dilution any saline or other ingredient through the skin or kidneys, is not different in its nature from any other effort to depurate the blood, when morbid agents have entered it or are retained within it.

I have stated, that in conscious animals the operations of the unconscious principle of intelligence are associated with a feeling of pleasure or well-being if normal, with a feeling of discomfort or suffering if abnormal. But I wish to include amongst conscious animals only man and the vertebrata; as to other organisms, it is as yet an open question whether they feel at all, or if they do feel, whether they feel both pleasure and pain. The phenomena of consciousness are only known to the consciousness. Doubtless the inferences which a man draws from his own experience, as to the feelings of other men, are in the main correct; and in admitting *mammals* and *birds* to brotherhood with him in respect to physical happiness and suffering, he is not far wrong; but it is not correct to lay down as a proposition, that a manifestation in organisms of the *external signs* of happiness and suffering usually manifested by himself, prove that the *feeling* of happiness or of suffering is experienced by them; or that such manifestations, and none other, are alone proofs. *Articulata* are popularly believed to feel acutely; plants are thought to be devoid of feeling altogether; yet the same class of phenomena are manifested by the latter as by the former, through the working of the unconscious principle of intelligence, the real difference being only in the organs and mode in and by which the phenomena are manifested. There is the same intelligent adaptation to circumstances; the same pre-arrangements for the same great objects; the same efforts for the conservation of the individual and the species under varying circumstances; and therefore fundamentally the same instincts. The difference is in the infinite variety of the means and modes. If we compare our own feelings with those of lower animals, we may reasonably admit that they at least enjoy life; for as to our *viscera,* the organs of vegetative life (which in them are pre-eminent), we have no other state of mind than a dim feeling of pleasant physical existence. When they are diseased or injured, we experience acute pain, not referred to anything external, and certainly more acute in proportion as we ascend from savage or uncultivated life, and much more acute, apparently, than in the lower vertebrata. But it is noteworthy, that the pain hardly dwells in our memories. Perhaps in the articulata there may be a dull sense of pain when injured, but no *memory* of pain; so that there is no *fear* of it; and what is felt is limited to the actual *moment* of injury. As to the vegetable kingdom, it is as reasonable an induction, that its members also enjoy life—possibly a *painless* existence—as that they have no consciousness whatever.

However this may be, it is quite certain, that in all conscious animals endowed with a nervous system, without any exception whatever, the special seat of both conscious and of unconscious mind is in that system, or in some part of it. Here, then, is something more than analogy, for there is identity. But since the development of the nervous system itself is the work of the unconsciously constructing principle of intelligence, and is formed by it with a special adaptation to the uses of the conscious mind, its structure does not fundamentally differ from the organized tissues equally so adapted which are devoid of nerves or nervous system. The contrary opinion is an error, which has broadly separated vegetable from animal organisms, and which has given rise to the hypothesis, that the lowest forms of the latter possess a "diffused" nervous system, microscopically small, or even invisible; it being a notion that the functions of these animals can only be carried on by something of the kind. It is now established, however, that these consist, like the analogous vegetable organisms, of simple cells. It follows, therefore, that the *protozoa* and *protophyta* constitute the *dynamical* types of the essential portion of the nervous system—the ganglionic cells in defined groups—or the vesicular neurine, in which the action is probably direct from cell to cell. The point of importance in vegetables is the division of labour amongst the cells, some secreting colouring matter; others, starch, gum, sugar, oil; and another the material for reproduction. Still, all combine to a common purpose—the well-being of the plant, and the continuation of the species.

In the higher animals, and in some vegetable organisms, the functions are more specialised, and are carried on by special apparatuses constructed for the purpose. Food is assimilated by one class,—is carried thus assimilated, to the molecular tissues by another; the results of waste and repair are various, and are carried off by various machinery adapted to the purpose; the germ-cells and sperm-cells are developed also in special tissues—the reproductive organs. There are also weapons for the defence of the organism; apparatus for the prehension of food, and for its mechanical division and preparation previously to assimilation; apparatus for the supply of the oxidizing material; apparatus for the *intus*-connexion of the sperm-cell and the germ-cell, &c. All these require to be combined in action for the attainment of the objects of the organism as a unity, and we have therefore a special apparatus formed for this end, in which that unconscious principle of intelligence, previously (and still, indeed) present alike in all cells, is now specially localized; this apparatus is the nervous system.

The *use* of these various machines and apparatuses, according to a predetermined and fixed plan, is termed *instinctive*.

We have already divided instinct into that which acts consciously, and that which acts unconsciously. Now instinct, in reference to cell-life, may also be divided into the individual and the composite. In the simpler forms of vegetable and animal life the individual existence is *perhaps* typified by the unicellular organisms; it is more certain that the higher animals which are evolved from a single cell are strictly *individuals*—that is to say, indivisible. The composite forms of vegetable and animal life—as yeast, hydras, the diplozoon paradoxon, the various compound entozoa, &c.—are perhaps rather *societies* of unicellular organisms than compound individuals. Be this as it may, it is in the organisms evolved out of a single cell, and in which all the separate organs are co-ordinated to the common object of the organism, that we have the first undeniable example of the *individuum*. Unity manifestly, therefore, precedes consciousness, and is, of necessity, the fundamental or primary idea of the unconscious principle of intelligence. If, then, there be a co-ordinating apparatus, by the operation of which all the separate organs are co-ordinated to the common object of the organism, it necessarily follows that that apparatus must constitute the centre of unity, or of the individual, and therefore the seat of the *ego*, if self-consciousness exist. This has been fixed hypothetically by some physiologists in the medulla oblongata.

Inasmuch as the nervous system, in virtue of its predetermined structure, is the source of the infinitely varied manifestations of intelligence in *action*, and the centre of co-ordination, so also is it the seat of that *great conservative idea*, for the attainment of which co-ordination takes place, inasmuch as the sole object of the entire arrangement is the well-being of the individual or of the species. Since what is true of the whole, is true of every part thereof, it follows that the nervous system is also the seat of all those *quasi*-mental or instinctive powers by which the unconscious mind attains its ends. Now, as the mind has, in summary, the same ends in view, it is absolutely necessary to inquire into the nature of these fixed arrangements of the vesicular neurine on which the instinctive acts depend, and their relations to consciousness.

It has been shown, that in the *construction* of the various necessary apparatus and instruments by which the great conservative idea is carried out into action, there is manifested a profound *knowledge* of numbers, geometry, mathematics, and of every department of natural philosophy; that is to say, all that the human mind knows of pure and mixed science (and, indeed, infinitely more) is *applied* to constructive art. If we investigate the working of the apparatus thus scientifically constructed, we find that they also are all *used* with an apparent similar knowledge. I refer more particularly to those instincts and instinctive actions in which either the *natural* instruments are used, exclusively and primarily, or else secondarily, for the *construction* of other means of conservation of the latter. No better illustration need be given than that familiar to naturalists, of the mathematical knowledge with which the domestic bee, as a formative artist, constructs its comb. The problem for solution is, to construct the cells with greatest strength, in the least space, and with the least expenditure of material—the daily problem of the human architect. Now this problem is solved by the bee, by selecting the hexagon as the geometrical form; by placing the cells base to base; and by causing the base of each to rest against the point where these partitions meet; thus saving materials and labour, and following out most exactly the principles of solid geometry.

It is a curious mathematical problem, Sydney Smith remarks, in his Lecture on the Faculties of Animals and of Men, at what precise angle the three planes which compose the bottom of a cell ought to meet, in order to make the greatest possible saving, or the least expense of materials and labour. This is one of those problems belonging to the higher parts of mathematics, which are called problems of *maxima* and *minima*. It has been resolved by some mathematicians, particularly by Mr. Maclaurin, by a fluxionary calculation. He has determined precisely the angle required; and he found by the most exact measurement the subject could admit that it is the very angle in which the three planes in the bottom of the cell of a honeycomb do actually meet. Of course, all this knowledge is no part of the consciousness or experience of the insect, yet it would take a senior wrangler at Cambridge ten hours a day for three years together to know enough mathematics for the calculation of these problems, with which not only every queen bee, but every undergraduate grub, is acquainted the moment it is born." I shall presently give an analogous illustration of the application of solid geometry by the unconsciously *constructing* mind to the construction of the *perfect* human form.

The *instinctive use* by the *individual* of the apparatus supplied to it ready made by the unconscious mind, has been always considered as something distinct from the *instinctive construction* of new or more fitting apparatus. From what I have already stated, it follows that there is no fundamental difference in the origin and nature of the two classes of phenomena; one or two illustrations will, however, set the matter in a clearer light. It is matter of common observation, that plants and animals are gradually adapted to any new external circumstances by *structural* changes in the organs of external relation. The leaves, *e.g.*, of the *Ranunculus aquaticus*, differ in structure according as they are above or under the water. If above, they become enlarged and simply lobed; if below, they are more finely cut. If, however, the plant, growing in a moist soil, is not overflowed, then the leaves are so developed, in adaptation to the new circumstances, that a new species, the *Ranunculus hederaceus*, is constituted. The same kind of adaptation to external circumstances is exhibited by almost every kind of animal; the more remarkable and obvious being those in which changes in temperature and climate have to be provided for. Thus, we have hair changed into wool in a cold climate, or wool into hair in a hot; so also the variations in the colouring matter of animals. These facts are familiar to naturalists, and are those which Lamarck has generalized into a system in his "Philosophie Zoologique." It is of importance to remember that this instinctive construction is not limited to changes in the leaves, limbs, &c., of organisms, but extends also to the co-ordinating apparatus, so that new instincts are developed in lower animals, and "habits," and new sources of pleasure in man. To this category may be referred, indeed, every phenomenon of this kind, including the acclimatization of animals and vegetables, the production of varieties by domestic culture, &c.

With the development of new vesicular arrangements, new apparatus, and new instincts, or instinctive actions, there is not unfrequently a repression, suppression, or deprivation of some of those which belong to the original type of the species. It is worthy of notice, however, that they are never absolutely eradicated; for when the appropriate stimuli (long absent from the race, perhaps) are again applied, the corresponding instinct reappears. As an illustration the following may be mentioned. The straw which has been used for bedding the *carnivora* in Wombwell's menageries is sold, and is capable of further use. Straw that had bedded the lions was made into bedding for some horses, and the latter immediately showed signs of alarm on entering the stable, snorting, snuffing, and trembling at the unwonted odour. Now it is certain that for many generations the English horse has had no experience of these his natural enemies, and his instinct of self-defence as regards them never exercised; yet the predetermined arrangement of the vesicular neurine in connexion with the sense of smell and the preservation from violent death was still there, and was duly brought into action so soon as the stimulus to which the arrangement is adapted was duly applied. Numerous similar examples of the persistence of these fixed arrangements might be adduced from the natural history of domestic animals, whether retained in the society of man, or passing again into a wild or half-wild life.*

As illustrative of the common source and nature of the instinctive use and construction of organs, I may mention changes in the colour, form, &c., of animals occurring under the immediate influence of instinct; as when concealment is desired, either to avoid enemies or seize prey. Insects, fishes, reptiles, birds, in numerous instances assimilate their colour to that of surrounding things, or change their colour (as the chameleon) in a moment. The loss and reproduction of limbs under the influence of the instincts belong to the same class of phenomena.

The habits of the solitary wasp, referred to by Sydney Smith, is an apt illustration of another point of view of this matter, inasmuch as it shows instinctive *action* in one form of organism taking the place of instinctive *construction* in others. In numerous animals, as well as in vegetables, the primordial cell is imbedded in a nutrient material contained within a shell or case, the whole constituting the egg or seed. The yolk of the egg (the nutrient part) is not only expressly adapted chemically to the wants of the growing animal, but is also exactly proportioned in *quantity*, so that when it is exhausted, the young being can either obtain food for itself, or is supplied by its parent. In mammals

* A sheep farmer has just stated to me an illustration of this principle, which I mention as showing the practical bearing of these views. Complaining of the loss of lambs he had experienced in consequence of the cold spring, I asked why he had not suitable lying-in hospitals constructed for the ewes, and he replied, one reason was, that only the Southdown (the highly-bred ewe) would submit to restraint. The ewe of the *Cheviot* breed, and of the black-faced or mountain sheep, *would* wander away to drop her lamb by herself, and was not easily restrained. The latter also display an impatience of being touched or handled by man, which the more civilized Southdown never manifests. Their semi-wild state on the mountain and moor pasture is clearly the source of these peculiarities.

the ovum is placed in the uterus, and is supplied by the circulating system of the parent with nutrient material. In many of the *hymenoptera* the whole business of the active life of the insect consists in the carrying out of these ends of the unconscious principle of intelligence. The construction of the case or receptacle for the ovum, and the filling it with provisions, manifest some of the most singular and interesting efforts of the reproductive instinct. As a special illustration may be mentioned that of the solitary wasp, which supplies to its ovum both a case and a suitable nutrient material. She digs several holes in the sand, in each of which she deposits an egg. Next (I quote Sydney Smith),

"She collects a few green flies, rolls them up neatly in separate parcels (like Bologna sausages), and stuffs one parcel into each hole, where an egg is deposited. When the wasp-worm is hatched, it finds a store of provisions ready made; and, what is most curious, the quantity allotted to each is *exactly sufficient to support it* till it attains the period of wasphood, and can provide for itself."

This instinct of the parent wasp is the more remarkable, as it does not feed upon the food it supplies to the ovum. An analogous instance of constructive development is seen in the economy of bees, when a queen or prolific female is wanted to be developed, and the bees supply certain larvæ with a special kind of food suitable to produce the required effect, the latter not being able to obtain it for themselves under the guidance of their own appetites. In short, it may be stated generally, that bees possess a power in the management of their offspring far beyond the power of man; for, by virtue of their instincts, they can develop them into males, females, or neuters, as the wants of their society demand. Strictly, a hive of bees is analogous to a composite animal, for these remarkable reproductive instincts are nothing else than the means by which the objects of the unconsciously *constructing* agent are specially attained in the individual. That which in vertebrates is secured by the laws of embryonic development, is attained in the hymenoptera (and indeed in *insecta* generally) by the instincts of the individual, or the society.

Another form of instinct remains to be noticed—namely, the adaptive direction of apparatuses and instruments already formed to the attainment of the wants of the individual under *new* circumstances. The class of acts thus caused have been designated *rational*, or adduced as instances of *reason*. They are, I think, not such in the common meaning of the terms. Mr. Gardner records, in his "Travels in Brazil," the following instance of apparent reason in a crab, a small species, belonging to the genus *Gelasimus*. It was either making or enlarging its burrow in the sand, and about once in every two minutes it came up to the surface with a quantity of sand enclosed in its left claw, which by a sudden jerk it ejected to a distance of about six inches. Mr. Gardner threw a small shell into its hole, others remaining within a few inches of it. In about five minutes the crab brought up the shell, and carried it to a distance of about a foot from its burrow. Seeing the others lying near the mouth of the hole, it immediately carried them one by one to the place where it placed the first, and then returned to its labour. In this and numerous similar instances, common to all animals, a higher manifestation of the unconscious soul is shown than occurs in those which are in immediate and direct dependence upon fixed arrangements in the vesicular neurine. It is the connecting link between instinct and reason; but it is not a manifestation of the knowing and willing *self*-conscious mind. In man, numerous similar acts are manifested during infancy and childhood.

It is obvious, then, that the unconscious soul, when constructing the coordinating apparatus, whether during development or in after-life, writes within it, as it were, its own principles of knowledge; and thenceforward the nervous system acts as wisely and as sagaciously as if endowed with mind, in all those actions which are independent of the will or the reason. The invariable sameness and permanence of the instinct in successive generations (the external circumstances being the same in each generation), and the transmission of acquired instincts and habits (the circumstances being different), constitute a strong argument in proof of the doctrine that they are dependent on special arrangements of the vesicular neurine—an argument confirmed by the numerous vivisections instituted to demonstrate the nature of reflex phenomena, all of which establish the fact, that *integrity of structure of the vesicular neurine* is the essential requisite to reflex movements. These special arrangements I have already designated the substrata of psychical phenomena.* These combinations or masses of nerve-cells are subject to the ordinary laws of quasi-mental action according to a fixed plan, whether they be formed during the life of the individual, or acquired by hereditary transmission; they have equally their appropriate stimuli, their appropriate progressive development, or their retrogressive change; and, singly or in combination, they may lead to the evolution of new masses of vesicular neurine, and new modes of mental action. Whatever may be their course, however, these arrangements of the vesicular neurine correspond in function (sensorial or motor) to the ideas, conceptions, and intentions of the unconscious mind. To the conscious mind of the organism their relation is wholly this—namely, that they enable it to attain to that which it desires, or to avoid that which it dislikes. If the appropriate stimuli be carried to the vesicular neurine and awake it into its proper functional activity, this vital machinery is duly put into operation. The corresponding change in the state of the consciousness is this, that if the stimuli reaching the vesicular neurine be in harmony with the modes of action writ upon it by the unconscious principle of intelligence, and changes follow in harmony with the objects it has in view, a feeling of pleasure is induced; but if the stimuli be not in correspondence with the fixed pre-determined mode of action of the vesicular neurine, and with the objects of the unconscious mind, pain or unhappiness results. This is, I think, an accurate general statement of the knowledge we have as to the relations of the inner working of its organ to the consciousness.

Our next step brings us into the field of human neurology and psychology. The unconscious soul of man, acting within the cerebrum, has its substrata—placed there *ab initio*, or constructed anew. What are they? and what are their relations to the consciousness? We shall find that the two forms of mental manifestation have a common origin and a common substratum, and that *the human mind is none other than the unconsciously working principle of intelligence individualized, becomes conscious of its own workings in the cerebrum, and deriving its ideas from its own constructive or material changes in the organ of mind.* This proposition I shall now proceed to demonstrate by a series of illustrations. First, as to consciousness itself.

The mind is One—a unity. "The unity of consciousness is at once the deepest, rarest fact of our nature, and the most rigid condition for a complete mental philosophy."†

This unity is to be found in the identity of the conscious and unconscious mind. I have already shown that, as regards the latter, the organism is an *individuum*, and that, therefore, unity is its primary idea and prime object. It is thus the *self*-conscious mind exists; its own existence as an *individual*—as a unit—implies the idea of its existence as a something distinct from everything else. This is its fundamental intuition or conviction. This conviction it retains so long as the co-ordinating apparatus within the cranium duly and

* On the Reflex Function of the Brain: § 3. The Substrata of Psychical Phenomena. "British and Foreign Medical Review," vol. xix. p. 308.

† Morell: "Elements of Psychology," p. 19.

normally fulfils its functions; if, however, these be interrupted, then the state of unconsciousness supervenes—or, in other words, consciousness (and there-with *self*-consciousness) is abolished. The exact locality in the encephalon which is the seat of consciousness—or, in other words, the centre of corporeal and mental unity—fixed by some in the medulla oblongata—is still undetermined; but that there is a central point, composed of vesicular neurine, in which the sum total of the functional activity of the organism is felt, and whence there is a reaction (reflex action) upon all the structures which minister to the physical well-being of the organism, is as certain as that every organism is *developed* from a common centre—the primordial cell.

Writers use the term *double* consciousness in reference to certain states of the mind in which the individual manifests, as it were, two distinct forms of mental life. A more correct term would be *alternating* consciousness, since it is most probable that the phenomena depend upon alternating independent action of each half of the cerebrum; but whatever may be the explanation, it is certain that the phenomena in question can never establish the doctrine of a *duality* of consciousness. Sir H. Holland appears to have set this point at rest.*

The unity of consciousness implies another fundamental principle—namely, that the varying states in which the latter exists are *successive*, and not contemporaneous. The mind cannot be occupied with two objects at identically the same moment. To assert the contrary proposition (a popular error) is to assert that the consciousness is divisible; whereas its unity implies its *indivisibility*.

"Sensation is not the object of consciousness different from itself, but is the *consciousness of the moment;* as a particular hope, or fear, or grief, or simple remembrance, may be the actual consciousness of the next moment. In short, if the mind of man, and all the changes which take place in it, from the first feeling with which life commenced, to the last with which it closes, could be made visible to any other thinking being, a *certain series of feelings alone*—that is to say, a certain number of successive states of the mind—would be distinguishable in it, forming, indeed, a variety of sensations, and thoughts, and passions, as momentary states of the mind, but all of them existing individually and successively to each other."†

I know of no inquiry into this part of mental physiology more lucid or more instructive than Sir Henry Holland's, and to his chapter On Mental Consciousness in Relation to Time and Succession, I would specially refer the reader.‡

The unity of consciousness implies another fundamental principle—that whatever changes in the vesicular neurine are presented to, or reach, the consciousness, and excite therein feelings, sensations, ideas or thoughts, are accompanied with a conviction of *truth* and *reality* as to the latter, whatever may be the source of the change; that is to say, whether it arise from morbid or healthy cerebral action.

"When we speak of the *evidence* of *consciousness,*" Brown remarks, "we mean nothing more than the evidence implied in the mere existence of our sensations, thoughts, desires, which it is utterly impossible for us to believe to be and not to be; or, in other words, impossible for us *to feel* and *not to feel* at the same moment."

Now, the ideas which are continuously and fixedly thus believed, in all normal states of the mind, are those termed *intuitive* truths, *innate* ideas, &c.

* "Chapters on Mental Physiology:" chap. viii., On the Brain as a Double Organ.

† Brown: "Lectures on the Philosophy of the Human Mind"—On Personal Identity.

‡ "Chapters on Mental Physiology," &c., p. 46 et seq.

They are dependent upon fixed and, in normal states of the cerebrum, unchanging arrangements and modes of action of the vesicular neurine; being such, they are writ upon the organism by the unconscious soul itself, are therefore its fixed and unalterable truths, and are to the human mind the *intuitions of pure reason.*

But what if the cerebral structure be disordered, either as to its vesicular arrangements, or its modes of action? Abnormal states of the consciousness will be induced; but, so long as consciousness exists, the mind will still feel convinced that the representations to the consciousness, which are presented in these disordered modes of action of the vesicular neurine, are real and true. The most common illustration of this fundamental principle is the state of the consciousness in dreaming, in which, as every one knows by personal experience, ideas the most absurd and the most incongruous as to time and space, are fully and indubitably believed. In artificial reverie, induced by the so-called electro-biological processes, an analogous state of the vesicular neurine and of the consciousness is induced; so also in artificially induced somnambulism, spectral illusions (clairvoyance), &c. In these the disordered action of the vesicular neurine is wholly functional and transitory; but in the delusions of the monomaniac they are permanent, and hence it happens that whenever that portion of the vesicular neurine which, in him, is the seat of the morbid action, is brought within the series of changes then being presented to the consciousness, the normal and therefore true succession of ideas is interrupted, and the abnormal and false occupy the mind fixedly, and, for some moments at least, to the exclusion of all others. This morbid presentation to the consciousness comes (like all others) with all the reality of truth, and, in proportion as it is continuous in time, it occupies the mind; for it is only by the constant succession of these changes in associated sequence, that erroneous ideas are corrected. Erroneous states of consciousness probably occur at many moments of our waking lives; not one of our senses is to be depended upon; but there is a pre-ordained mutual control and correction of each other in healthy action, which is destroyed in dreaming and other abnormal states of the cerebrum. The detection of monomaniacal delusions is sometimes difficult, because the patient, being keenly conscious of his infirmities, will conceal them; if, however, by what is termed the association of ideas, the morbid action of the vesicular neurine be brought within the current of his thoughts, he becomes utterly powerless to resist it—as much so as the electro-biologised to resist the suggestions presented to their minds. The formation of these monomaniacal *substrata* is due (as all observation shows) to the fixity of the mind on one idea, or class of ideas, at a time when, from morbid changes induced in the vesicular neurine (as by undue mental labour, intense emotional excitement, want of repose, the development of a dormant predisposition, and the like), it is unusually susceptible of the operation of the unconsciously constructing mind; so that the fixed ideas become deeply writ, as it were, on the vesicular neurine, just as acquired instincts, habits, &c.; and are, in fact, as difficult to remove.

The intuitive conviction of *continuous* existence in time and space, known as the feeling of *personal identity*, has a more complex origin than is usually laid down. It implies two fundamental requisites—namely, a *perception* of the external world and *memory*, together with all their dependent faculties and modes of action. In that state of the consciousness which is a feeling simply of pleasure or of pain, there is no reference to the external world; in the higher state of *self*-consciousness, there is the latter necessarily, because the unconscious mind provides, by its inner vesicular arrangements, for the external world. It not only aims at the well-being of the organism, but provides, by its predetermined plan of construction and action, for the acquisition from without of what is beneficial, and the expulsion or repulsion of what is obnoxious. This is what the unconscious mind aims at; it follows, therefore, that as the conscious mind it desires them. The completion of the desire is accompanied by a feeling of pleasure, inasmuch as that completion is in congruity with the predetermined arrangements of the unconscious mind, which feeling is termed *satisfaction*, joy, pleasure. The desire to attain the good is usually termed *desire*, simply to avoid the evil is termed *abhorrence*. Now just as the unconsciously constructing principle of intelligence adapts the inner vesicular arrangements to external circumstances in plants and in the lower organisms, and so develops new instincts and instruments, so also, during the operations of the conscious mind, it constructs or arranges the vesicular neurine in accordance with its operations. These changes, whenever they are such that they can be presented to the consciousness, will come within the continually flowing series of states of the latter, which constitute the sum of mental existence; and being thus the unconsciously-written record in the vesicular neurine of the successive operations of the mind, constitute the material *substrata of memory*. The substrata, therefore, of acquired instincts, habits, &c., and of memory, are due to a common cause and common mode of action; the former, when transmitted, constitute, in fact, the *memory of the species;* the difference is in the relation of the respective substrata to the states of consciousness, and its relations to the external world.

It is not possible to comprehend the phenomena of memory without the concession of the doctrine, that the mind thus working unconsciously, continually constructs or arranges the vesicular neurine of the cerebrum. In his lucid chapter, "On the Memory as affected by Age and Disease," Sir Henry Holland mentions several interesting illustrations of the general fact—"That, of all the intellectual powers, it depends most on organized structure for whatever concerns its completeness, its changes, and decay," but has strongly experienced the absolute insufficiency of all theories founded on the connexion of memory with organization to explain several of its phenomena. It is, perhaps, in the doctrine I have just advanced that a more satisfactory explanation may be found. These substrata of memory are essential to the feeling of personal identity—i.e., of continued existence in time. The idea of continued existence includes the ideas of the past and the future. It is an intuition that we shall continue to exist, as well as that we have existed. Now this idea of the future is a fundamental idea of the unconscious principle of intelligence—equally fundamental as the idea of unity itself. Its aims and acts are all, without exception, *prescient;* the continued existence—i.e., the existence in time to come of the individual or of the species—is its great object. Hence, the infinite variety of prescient instincts displayed by all organisms, whether animal or vegetable; hence the instinct for continued existence, or love of life, and the universal abhorrence of death; hence it is that "men think all men mortal but themselves." In desire, the idea of the future is necessarily involved, whether it is a good we desire to acquire, or an evil we desire to avoid. The desire realized is the present, often too quickly to become a thing of the past.

Morbid conditions of the vesicular neurine develop correlative states of the consciousness in reference to these fundamental intuitions. *Neuralgia*—i.e., an ache or pain, simply dependent on a morbid state of a nerve or a ganglion of common sensation, and constituting a modification of the primary form of consciousness—is one. *Melancholia* is a higher morbid state in which evil is anticipated, or believed to have occurred; it is, however, precisely analogous to neuralgia in its nature. In the kind of dreams in which everything goes wrong, and in "low spirits," when all kinds of anxious fears are experienced, we have a condition analogous to the condition of the vesicular neurine in melancholia, only in the latter the condition is permanent, in the former it is transient. Melancholia has been termed phrenalgia by Guislain, and in one sense the term is correct; it is a term of doubtful meaning, however, for it may imply that the sources of the states of consciousness grouped under the term are in the mind itself; whereas they spring from morbid modifications of the vesicular neurine. The state of consciousness induced is precisely antagonistic to the aims and objects of the principle of intelligence, which is happiness, and to that experienced in the normal condition of the neurine: hence it is, that things pleasurable naturally become changed in their effects: rightly, therefore, the melancholic Hamlet says of the highest source of natural pleasure—"This most excellent canopy, the air; look you, this brave o'erhanging firmament, this majestical roof fretted with golden fire, why, it appears no other thing to me than a foul and pestilent congregation of vapours." In the same way it is that, in neuralgia, impressions ordinarily agreeable—as of light, sounds, touches—are the sources of acute pain.

Neuralgia, in its primary and simplest form, is pain only; but there are forms in which there are painful illusive sensations, as of pricking, stinging, burning, coldness, &c.; in these there is a reference to a cause external to the organism. Closely related to these, are the illusions of the hypochondriac as to his bodily sensations, and as to the morbid states of his viscera: and in intimate connexion with these latter are those morbid states in which there are delusions as to what may be termed the anatomy and intimate construction of the body or its parts. Thus, melancholic patients will assert that they have no stomach, no bowels, no head, no soul; that they, or some portions of them are made of butter, glass, or something else easily destructible. They will have delusions as to their personal identity, as to their preservation in general (fear of death, vague apprehensions); or as to their danger from particular sources of injury (suspecting melancholia). Now, just as in neuralgia there is a complete perversion of the predetermined respondence to impressions, so in melancholia there may be a complete perversion of the predetermined instincts and modes of thought; and the trembling melancholic—who expects and dreads his death, flies from the most trivial things, in terror of death at every moment—becomes profoundly suicidal. The transition from a morbid condition of this kind to that in which the active instincts of defence are roused, is a natural and not unfrequent occurrence, so that the suicidal is often a homicidal maniac; or else the nutrient instincts are involved, and the hypochondriacal dread of being poisoned passes into the maniacal determination to take no food, or to take poison. This doctrine of the pathology of melancholia is equally applicable to all forms of the disease.

The preceding illustrations of the relation which the instincts and emotions bear to the vesicular neurine, and through it to the unconscious principle of intelligence, are, I trust, amply sufficient to show the exact correlation between the latter, and conscious mind in all modes of thought and states of consciousness in which the instincts, emotions, and passions, are predominant. I will now submit illustrations taken from the domain of the intellectual powers, and will select two points of special and comprehensive importance — namely, reason, or intelligence, itself, and intellectual pleasure, or happiness.

An act of the reason implies a knowledge of the qualities of matter; the primary idea, therefore, of the intelligence, must be the intuitive idea that matter exists. Now, the external world, and the qualities of matter in relation to the organism, constitute the study, if the phrase may be permitted, of the unconscious mind; correlatively, therefore, these are the study of the conscious mind. The first rise of the *ego* of self-consciousness is in the perception of that which is not a part of the individual, or external to it. The body is a unity that it may be the more effectually protected from external injurious agents, and secure its well-being and the happiness of the soul which it clothes. The evolution of all the apparatuses and instruments of sense, in particular, has the special end in view of placing the seat of unity and consciousness in instantaneous and intimate communication with the external world, through what may be termed prolongations, or projections outwards, of the vesicular neurine; for the nerves of special sense are virtually nothing else than portions of the grey matter spread out on apparatus suitably constructed for the reception of the influences which matter can exercise upon the vesicular neurine of the cerebrum, itself also especially arranged for being influenced by them. All the nerves, therefore, of special sensation at least (or, in other words, *all* sensory nerves, exclusive of those which minister to pleasure and pain only), have a common function and common principle of action. They may be considered as nerves of touch. This being the fundamental aim and method of the principle of intelligence, it follows that all changes in the consciousness consequent upon changes in the sensorial ganglia are accompanied with the conviction that the sensations arise externally. As to tactile impressions, this may appear of doubtful application; but it must be remembered, that the entire body is external to the consciousness. It is probable, that in a perfect act of perception all the senses co-operate in the act, and erroneous ideas are prevented by that predetermined mutual control and combination to a given end which I have already referred to as part of the function of the vesicular neurine. In morbid states of the latter, as in neuralgia of a stump, the mind refers the seat of pain to a point altogether apart and external to the true seat, because there is no provision for a correction of the impression. In auditory or visual illusions, dependent on cerebral disease, the same result is observed if the person be insane; or, in other words, if the cerebrum be so disordered that the necessary correction cannot be made. This idea of *outness* is fundamental to all perceptions.

The ideas of *power* and of *causation* (or cause and effect) arise in the mind in the same way. We have seen that it is the aim or idea of the unconscious agent, in laying down the predetermined arrangements of the organization, that they shall invariably respond to the same stimuli; this idea is reproduced as a state of the consciousness, and is the idea that they will, for the future, so respond:

"Why is it, then," says Brown, "that we believe in that continual similarity of the future to the past which constitutes, or at least is implied, in our notions of power? A stone tends to the earth—a stone will tend to the earth—are not the same propositions, nor can the first be said to involve the second. It is not to experience, then, alone that we must have recourse for the origin of the belief, but to some other principle, which converts the simple facts of experience into a general expectation or confidence that is afterwards to be *physically* the guide of all our plans and actions. This principle, since it cannot be derived from experience itself which relates only to the past, must be an original principle of our nature. There is a tendency in the very constitution of the mind, from which the expectation arises—a tendency that, in everything which adds to the mere facts of experience, may truly be termed instinctive." (Op. cit., vol. i. p. 121.)

When a stimulus or impression has excited the functional activity of any predetermined arrangements of the vesicular neurine, to which it is adapted, the state of consciousness corresponding thereto is correlative with the idea of the unconscious principle of intelligence; now it is the aim of the latter that that effect should be so produced invariably, consequently that which invariably precedes a change in the state of the consciousness is connected in the mind with the idea of a *cause;* hence the idea of *causation.* Thus Brown:

"A cause is, perhaps, not that which has merely once preceded an event, but we give the name to that which has always been followed by a certain event, and, according to our belief, *will continue* to be in future followed by that event, as its immediate consequent; and causation, power, or any other synonymous words which we may use, express nothing more than this permanent relation of that which has preceded to that which has followed . . . . To know the *powers* of nature is, then, nothing more than to know what antecedents *are* and *will be* invariably followed by what consequents." (p. 120.)

This is, in fact, the foundation of all science. Nature is nothing else than the predetermined arrangements in operation of the great creating and sustaining Intelligence, which it is the duty of man, a "*naturæ minister et interpres*" to know. The faculty by which he ascertains these invariable relations of phenomena to each other, is termed *comparison.*

I could thus go through all our fundamental ideas and all our intuitive truths, and show that in them all the states of consciousness of the self-conscious mind are correlative with the ideas manifested in organization by the unconscious mind; and that it is from the manifestations of the latter in and through the functional activity of the predetermined arrangements in the vesicular neurine, that all thoughts arise into our consciousness. There can be no doubt whatever, whether we consider the deductions to be drawn from observations of the form of men's crania, from the investigations of pathology and pathological anatomy, from the facts of comparative anatomy and zoology, and from the laws of embryology, or whether we consider the general laws of psychology as displayed in the operations of the unconscious mind—that, just as there is a differentiation in the tissues and structure of the body, to secure its well-being and continuance,* so also there is a differentiation in the co-ordinating apparatus itself, to secure a knowledge of the external world. The result of this is a constant localization and specialization of function, so that masses of vesicular neurine are progressively appropriated to the mental powers as they are evolved, extent of neurine being correlative, *mutatis mutandis,* with extent of manifestation of the power. In these masses there is the same fixed respondence to the appropriate stimuli, as in the ganglia with simpler endowments; the same correlation between the ideas of the unconsciously constructing mind and the consciously thinking mind; and the same relation between the appropriate respondence to stimuli of the neurine and the states of consciousness known as pleasure and pain. The fundamental modes of action of the human mind and its organs are really, therefore, INSTINCTIVE.

It is a remarkable circumstance, that while metaphysicians and phrenologists have alike almost unanimously advocated or adopted this doctrine, it has never been applied to the elucidation of the nature of mind, by constituting it the starting point of a *comparative* psychology.†

* See Dr. Carpenter's "Principles of Comparative Physiology," fourth edition, pp. 18—20, 38, for a statement and illustration of this fundamental process.

† I subjoin the following rather long extract from Sir W. Hamilton's Note A (p. 761), in his "Dissertations," &c., supplementary to his edition of "Reid's Works," on account of the vast importance of this doctrine to mental physiology and pathology: "An instinct is an agent which performs blindly and ignorantly a work of intelligence and knowledge. The terms *instinctive belief—judgment—cognition,* are, therefore, expressions not ill adapted to characterize a belief, judgment, cognition, which, as the result of no anterior consciousness, is, like the products of animal instinct, the intelligent effect of (as far as we are concerned) an unknowing cause. In like manner, we can hardly find more suitable expressions to indicate those incomprehensible spontaneities themselves, of which the primary facts of consciousness are the manifestations, than *rational* or *intellectual instincts.* In fact, if Reason can be justly called a developed Feeling, it may with no less propriety be called an illuminated Instinct—in the words of Ovid—

'Et quod nunc Ratio, Impetus ante fuit.'

As to [Reid's use of the term being] an innovation either in language or philosophy, this objection only betrays the ignorance of the objector. Mr. Stewart ("Essays," p. 87, 4to edition) adduces Boscovich and D'Alembert as authorities for the employment of the terms Instinct and Instinctive, in Reid's signification. But before Reid he might have found them *thus* applied by Cicero, Scaliger, Bacon, Herbert, Descartes, Rapin, Pascal, Poiret, Barrow, Leibnitz, Musæus, Feuerlin, Hume, Bayer, Kames, Reimarus, and a host of others; while, subsequent to the 'Inquiry into the Human Mind,' besides Beattie, Oswald, Campbell, Fergusson, among our Scottish philosophers, we have, with Hemsterhuis in Holland, in Germany Petens,

I will now examine into the source and conditions of *intellectual pleasure* in relation to the cerebrum, taking as a starting-point the doctrine that this organ is the seat of the intellectual instincts. It is necessary to the manifestation of these instincts in consciousness, that is to say, in thought and knowledge, that there be a predetermined arrangement of the vesicular neurine—psychical substrata—corresponding to each, so that when the appropriate stimuli reach it, the corresponding states of consciousness (or sequences—associations—of ideas) may follow. It is necessary to the *perfect* manifestation of these instincts that the aims, conceptions, or ideas of the unconscious mind be writ within the vesicular neurine. Now, we have seen that these are founded upon a profound (perhaps perfect) knowledge of the laws of matter, whether they be physical, chemical, or vital; it is, therefore, a necessary inference that the human cerebrum is, *potentially* at least, the seat of this knowledge; or, in other words, that by a suitable development of the material substratum, through the agency of the unconscious mind, the human mind *may* attain to this knowledge to a greater or less extent, and that the elementary principles of all branches of science may be more or less innate or intuitive. We have seen how the bee is an intuitive builder according to the most correct mathematical formula, in virtue of the same properties which we would assign to man. Now, the first instinct of human nature, and perhaps the highest intellectual pleasure, is to seek after and attain to knowledge*—knowledge of the world around him, knowledge of himself, knowledge of his relations to his Creator and his fellow-creatures. He is ever endeavouring to know the order of nature, or the causes of things—i. e., what is the necessary antecedent to a consequent; for it is knowledge only which gives him the freedom he continually strains after, and the dominion over matter he would conquer. *Felix qui potuit rerum cognoscere causas* is the sentiment of every man. This general use of the intellectual faculties, and the happiness consequent on the right use, is strictly analogous to that general use of the corporeal organs which constitutes the sum of life, and is, when normally carried on, the source of the feeling of corporeal happiness.

The unconscious principle of intelligence, as a constructive agent, aims not at the good only—τὸ εὖ; ever conjoined therewith is the beautiful—τὸ καλῶς. In the conscious mind this aim at the beautiful becomes a *desire*, when the vesicular neurine is appropriately evolved. Hence it is that amongst the special intellectual pleasures of which man is capable of feeling, are those derived from the fine arts—namely, music, painting, sculpture, architecture, and formative arts generally. These arts being practised by the unconscious mind in the construction of organisms, and in the instincts of lower animals, they present the best subjects for comparison and elucidation. Perhaps the human form may be reasonably assumed as the form the contemplation of which (when perfect) gives the highest intellectual pleasure. It may be considered under two aspects, first as constructed by the principle of intelligence; secondly, as constructed by man. According to the doctrine I wish to establish, the psychical substrata (the work of the unconscious mind), by and

Jacobi, Bouterweck, Neeb, Köppen, Ancillon, and many other metaphysicians, who have adopted and defended the expressions. In fact, Instinct has been for ages familiarized as a philosophical term in the sense in question—that is, in application to the higher faculties of mind, intellectual and moral. . . . . In a moral relation, as a name for the natural tendencies to virtue, it was familiarly employed even by the philosophers of the sixteenth century . . . . and in the seventeenth it had become, in fact, their usual appellation."

* There is an admirable little work on this subject, to which I would specially refer the reader, and the more earnestly because its value is not generally known—Sir John Forbes' "Happiness in Relation to Work and Knowledge: an Introductory Lecture," &c. Smith, Elder, and Co., Cornhill; or Churchill.

through which the beauty of the human form is felt and perceived, will be correlative with the constructive ideas and conceptions of the unconscious principle of intelligence (or nature, as it is usually designated); so that when the visual impression of a perfect human form reaches substrata perfectly evolved, there is congruity between the latter and the former; and the resulting changes in the consciousness in reference to the visual object are accompanied by that change in the consciousness termed pleasure. The same doctrine applies equally to all artistic impressions derived from the results of true formative art, whether seen in vases and objects of virtû, or in the grander architectural products of human genius; to all æsthetic combinations of colour; to the infinite variety of sweet concords. The recipient senses having an analogous structure, and a common function in relation to consciousness, the ideas that enter the mind through them have a common relation to the feeling of pleasure.

These substrata will also regulate the successive states of consciousness in relation to the objects of intellectual pleasure, and through them, therefore, it is that the mind conceives, either instinctively (as genius), or deductively through knowledge, correct conceptions of those objects; and realizing these conceptions, works matter into artistic forms, harmonizes colour, or combines sounds; which results are perfect accordingly as they approach the model or archetype in the unconscious mind.

The human female, in the perfection of youth and beauty, is to man probably the most beautiful, and the most pleasurable, visual object in creation. Often, doubtless, the artistic feeling of pleasure is associated with the instinctive feeling: but many of my readers will agree with me in the statement that the one is often unalloyed by the other; and that an abstract perception of the beautiful is excited by this example of the artistic perfection of the constructing principle of intelligence. The physiologist can trace visually the formation of that example from the union of sperm-cell and germ-cell, constituting the primordial cell, to its complete evolution at puberty; and he sees nothing more, in any part of the process of formation, than a combination of cells, according to fixed never-varying rules—or, if varying, leading to imperfect results. To him the fundamental form is a hollow spheroid, or ellipsoid, or a combination of such; the fundamental process a constant combination, re-combination, and multiplication of them. Now, the geometrical rules by which these histological elements are finally combined together, or collated, by the unconscious mind into a form of beauty, appear to have been determined by Mr. Hay, of Edinburgh, who has been sedulously labouring for many years past to elaborate the true principles of beauty in formative and decorative art, just as the geometrical rules by which the bee constructs its hexagonal cells have been determined by Maclaurin. These rules are based on a law of harmonic ratio, "identical," Mr. Hay remarks, "with that by which, through the organs of hearing, the mind is æsthetically impressed with one of the most refined and delightful emotions which mere sensation is capable of exciting, and on which are necessarily based the fundamental principles of musical composition." Mr. Hay lays down, as his first position, that the eye is influenced in its estimation of spaces by a simplicity of proportion, similar to that which guides the ear in its appreciation of sounds; and, as his second, that the eye is guided by direction rather than by distance, just as the ear is guided by number rather than magnitude of vibrations. The basis of his theory is simply this:—"That a figure is pleasing to the eye in the same degree as its fundamental angles bear to each other the same proportions that the vibrations bear to one another in the common chord of music." As to these vibrations, we quote Mr. Hay on the sounds of the monochord.

"This is an instrument consisting of a string of given length stretched between two bridges standing upon a graduated scale. Suppose this string to be stretched until its tension is such, that when drawn a little to a side, and suddenly let go, it would vibrate at the rate of sixty-four vibrations in a second of time, producing, to a certain distance in the surrounding atmosphere, a series of pulsations of the same frequency. These pulsations will communicate through the ear the musical note literally signified by C, which would, therefore, be the fundamental note of such a string. Now, immediately after the string is thus put into vibratory motion, it spontaneously divides itself into two equal parts, the vibrations of each of which occurring with a double frequency—namely, 128 in a second of time, and consequently producing a note doubly acute in pitch, although much weaker as to intensity or loudness ; that it will then, while performing these two series of vibrations, divide itself into three parts, each of which vibrating with the frequency triple that of the whole string—that is, performing 192 vibrations in a second of time, and producing a note corresponding in increase of acuteness, but still less intense than the former ; and that this continues to take place in the arithmetical progression of 2, 3, 4, &c. Simultaneous vibrations, agreeably to the same law of progression, which, however, seems to admit of no other primes than the numbers 2, 3, 5, and 7, are easily excited upon any stringed instrument, even by the lightest possible touch. The musical sounds thus naturally produced are called the Harmonics. . . . . The musical note produced by the vibratory motion of the whole length of such a string is, as I have already stated, called (C), and is, consequently, the fundamental note or tonic to which all that follow in forming a scale must refer. The note produced by half of the string is the first harmonic, and is called the superior octave to the fundamental note."*

Now all solid bodies are referred to plane figures upon the retina, and are bounded either by curves or right lines. If by the latter, their outlines are portions of rectilinear figures; if the former, of circles, ellipses, &c. Each rectilinear plane figure has a curvilinear figure that belongs to it—that is, a figure which may be symmetrically inscribed within it; and since every rectilinear figure may be reduced to a triangle, and a triangle is measured by its smallest angle, so also may curvilinear figures be measured by the angles of the rectilinear figure to which they belong. The theory of the pleasing in form being "that the division of space into an exact number of equal parts will æsthetically affect the mind through the medium of the eye, in the same way that the division of the time of vibration in music into an exact number of equal parts æsthetically affects the mind through the medium of the ear," it follows, that the first step in demonstration is to show the correlation between the fundamental *notes* and fundamental *spaces*. Two straight lines cutting each other—that is to say, a perpendicular and a horizontal line—form at their junction a right angle; and if they be equal in length, and their points be joined by a curved line, equally distant at all points of the curve from the angle of junction, the curve measures one-fourth of a circle, or 90°. The angle (a right angle) is therefore an angle of 90°. This quarter circle corresponds to the monochord in Mr. Hay's theory, and is divided by him in the same numerical ratio that the vibrating monochord divides itself, as just explained; the result being a series of rectilinear and their corresponding curvilinear figures, measured by the angles thus produced, correlative with the fundamental notes. When the parts or vibrations that constitute a musical sound are multiples of the fundamental number by 2, 4, 8, &c., they are called *tonics;* by 3, 6, 12, &c., *dominants;* by 5, 10, 20, &c., *mediants*, by 7, 14, 28, &c., *sub-tonics*. So in plane figures. Divisions by 2, 4, &c., give tonic angles; by 3, 6, &c., dominant angles; by 5, 10, &c., mediant angles; by 7, 14, &c., angles of the seventh degree, or fundamental discord. These angles may be also represented by figures, thus: 90° being taken as 1, an angle of 45° is an angle of $\frac{1}{2}$; 30° of $\frac{1}{3}$; 22° 30′ $\frac{1}{4}$; 18° of $\frac{1}{5}$, and so on. There is, therefore, a scale of harmonical angles exactly corresponding to a scale of harmonical notes; this Mr. Hay

* "The Geometric Beauty of the Human Figure Defined," &c., 4to, pp. 6, 7. 1851.

gives.* The tonic, dominant, and mediant notes produce, when combined, the most beautiful harmony; correlatively, the geometrical figures and forms of which the tonic, the dominant, and the mediant angles are the primary elements, are also the most beautiful of their kind. These views Mr. Hay applies to the Parthenon, to the leaves of trees, to flowers, and to the human form. Illustrations of these are given in the last-quoted work. His views have also reference to the identity of the laws of intellectual pleasure derived through the senses, quoting as to this principle a hypothesis of Sir Isaac Newton, thus expressed: "I am inclined to believe some general laws of the Creator prevailed with respect to the agreeable or unpleasing of all our senses; at least, the supposition does not derogate from the wisdom or power of God, and seems highly consonant to the simplicity of the macrocosm in general."

To construct the human female form in perfect proportion, Mr. Hay takes the first eleven harmonic angles as they arise consecutively from a division of the right angle, which he adopts as the fundamental angle, and combines them geometrically. First he lets fall a perpendicular line, representing the height of the figure to be constructed, and from this line draws his angles, according to a system only to be understood by a reference to his treatises. The curves of the figure are portions of circles and ellipses, whose angles of inclination are simply those of $\frac{1}{2}$, $\frac{1}{3}$, $\frac{1}{4}$, $\frac{1}{5}$, $\frac{1}{6}$. The following is Mr. Hay's summary:

"1st. That on a given line the figure is developed as to its principal points entirely by lines drawn either from the extremities of this line, or from some obvious and determined localities. 2nd. That the angles which these lines make with the given line, are all simple multiples or sub-multiples of some given fundamental angle, or bear to it a proportion admissible under the most simple relations, such as those which constitute the scale of music. 3rd. That the contour may be resolved into a series of ellipses of the same simple angles; and fourth that these ellipses like the lines, are inclined to the first given line by angles which are simple multiples or sub-multiples of the given fundamental angle. . . . . Thus there is a perfect harmony of combination in its proportions, associated with as perfect a harmony of succession in its beautifully undulated outline, the curves of which rise and fall in ever varying degree, and melt harmoniously into one another like the notes of a pleasing melody. When, therefore, we reflect that the scientific investigations of the anatomist have proved, that in the fitness of its parts the construction of the human frame exhibits the closest approximation in nature to a perfect development of mechanical science, and that similar investigations of the physiologist have proved that the processes by which it is sustained in vital energy exhibit the closest approximation in nature to a perfect development of chemical science, it cannot in any way be surprising to find that, in like manner, and agreeably to a definite and acknowledged law, the beauty of its form discloses the nearest approximation in nature to a perfect development of the science of æsthetics."†

Through Mr. Hay's kindness I am enabled to give a woodcut, with the angles upon it, from a drawing taken by Mr. Houston, R.S.A., of a Scottish female employed in the Royal Scottish Life Academy as a model. All the points of this figure correspond, except the hands, which are a little larger (probably from hard work), and the waist, which has evidently been compressed by stays, with the theoretical figure. The real variation is in the national high cheek-bones and broad Scottish face of the living model. Professors Kelland and Goodsir also assisted Mr. Hay in carefully measuring six living models, the classic statue known as the Mediceau Venus, and another as the Venus of Melos. The results corresponded so closely with the theory as to leave

* "The Orthographic Beauty of the Parthenon," p. 21; also, "The Geometric Beauty of the Human Figure Defined; to which is prefixed a System of Æsthetic Proportion." Appendix.

† "The Natural Principles of Beauty as Developed in the Human Figure," by D. R. Hay, F.R.S.E., p. 23.

J.A. HOUSTON R.S.A.

no doubt of its accuracy as to the living model, and to render it probable that a similar system constituted the basis of artistic education among the ancient Greeks.*

Fitness, strength, and beauty, are combined in the constructions of the unconsciously constructing principle of intelligence; these are the objects to be aimed at in architectural and the other formative arts. In one of his recent works† Mr. Hay demonstrates, by numerous measurements, that one of the most beautiful structures of antiquity, the Parthenon at Athens, was constructed on geometrical harmonies identical with those according to which the perfect human figure is developed or formed. The right angle (90°) is the fundamental tonic; taking this as the key-note, Mr. Hay theoretically re-constructs that grand architectural harmony throughout all its details; and then shows that the actual measurements correspond sufficiently near to the theoretical to demonstrate their identity.

In the application of geometrical ratio to architecture, Mr. Hay has had numerous predecessors; it is in selecting *angular* proportion as the basis of his harmonic system, instead of linear, and in applying his principles to curvilinear as well as rectilinear figures (especially the composite ellipse), that he differs from them. Nevertheless, the geometrical harmonies derived from *linear* proportions have an extensive application, especially to Gothic architecture. In these the *three primary forms*—the equilateral triangle, the square, and the pentagon—are fundamental figures.

Mr. Griffith (who has illustrated this theory) terms the governing figure in his system of numerical rectilinear ratio, the *kleis* (κλεὶς, *clavis*, key), but he draws his analogies from chromatics rather than acoustics, and makes his three primary forms analogous to the three primary colours—yellow, red, and blue. The system not only evolves all the ornamental details as well as the ground-plan, but also the greatest strength and elevation; for the same geometrical lines which dictate the latter "indicate the direction of all the thrusts or forces, and their sundry workings."‡ The ratios in the rectilinear system are the same as in the angular; and curvilinear figures are deducible from the rectilinear.

In another work, published in 1845, entitled "The Natural System of Architecture," in which the theory is applied to both Greek and Gothic structures, Mr. Griffith examines and delineates the geometrical proportions of the following Greek temples (amongst others), the Parthenon, Erechtheion, the Temple of Bacchus at Teos, of Themis at Rhamnus, and of Theseus. Amongst the Gothic structures are York and five other English cathedrals; and the Temple Church and King's College Chapel, amongst minor examples. Writers since Griffith have also taken up this subject, but on the same principles. We may infer, therefore, that the changes in the vesicular neurine, occurring during consciousness, have a definite relation to geometry and dynamics.

The views just advanced apply exclusively to the absolutely beautiful and true. Pleasure may be derived, however, from that which is relatively beautiful and true; and, indeed, this is the most common source of our pleasure. All special substrata, acquired either by inheritance or by the external relations of the individual, do modify the states of consciousness by the changes going on within them, when the appropriate stimuli reach them. To the former belong secret, "occult," or mysterious sympathies; to the latter the pleasures

* In another and earlier work (1849), entitled "On the Science of those Proportions by which the Human Head and Countenance, as represented in Works of Ancient Greek Art, are distinguished from those of Ordinary Nature," 4to, Mr. Hay treats fully of this subject.

† "The Orthographic Beauty of the Parthenon referred to a Law of Nature." 1853.

‡ "Ancient Gothic Churches, their Proportions and Chromatics." By William P. Griffith, Architect. Part II. p. 21.

of memory. Thus it is, that in a foreign land to hear the familiar language of home is a delight, or even to experience any impressions associated with pleasurable feelings felt at home. It is in confounding these different sources of pleasure, indeed, that the greatest obstacle to a true system of æsthetics and a sound philosophy of morals exists.

Having thus shown the instinctive *nature* and *origin* of our intellectual faculties, I shall now illustrate their instinctive *action*. It has been seen that acquired knowledge is no essential part of instinct generally, neither is it of these faculties when working instinctively. That which *is* necessary is a full development of the psychical substrata appropriate to each, or phrenologically the cerebral "organ." Persons endowed with these, and who have put them into action so as to evolve results, are known by the term *Genius*. Functional *activity* is, however, necessary; that is to say, in all artistic conceptions there must be the power to *represent* either to the eye or the ear. Most men who observe the working of their own minds, are cognizant of a power to conceive far beyond a power to execute; whether it be to clothe their ideas in appropriate language, with due rhythmical cadence (of which poetry is but one form), or in appropriate combinations of musical sounds, or in the visual music and rhythm of sculpture and architecture. Often the power to execute is greater than the power to conceive; thus, persons who know not a musical note, will play on the piano any tune which they have heard once or twice. Mozart is an example of true musical genius. When only four years old he began to write music in strict accordance with the rules of musical composition, although he had not been instructed in them. In after life he wrote music because, to adopt his own expression, he could not help it. So it was with an eminent English poet:

"I lisped in numbers, for the numbers came."

Instances of this kind could be multiplied to an almost indefinite extent.*

An illustration of the instinctive working of the *numerical* faculty may be added, to show that the doctrine is generally applicable. Mr. Roby, a banker at Rochdale, played, sang, composed, and was an amateur painter. His most developed intellectual instinct however, was his powers of calculation, in which he was superior to Bidder, perhaps the most wonderful calculator this country ever produced. His widow states in his published "Remains," edited by her:

"If a double column, twenty figures in each row, or a cube of six, were placed before him, he would tell the sum as soon as his eye could read the figures. He arrived at the result without going through the ordinary process; he saw it at a glance. If, as was rarely the case, owing to a passing fit of dulness, or a momentary distraction of thought, he failed to see the sum at once, he was rather slow than otherwise in doing it by the ordinary mode."

* Much knowledge might be gained from a careful observation of the instinctive working of these faculties. The following is an interesting fact taken in connexion with the preceding statements; it is from the "Diary of Moore," the poet (edited by Lord J. Russell, vol. ii. p. 237): "Dined at Power's, to meet Bishop, the composer, who is one of the very few men of musical genius England can boast of at present. . . . The omission of the seventh and fourth, he says, is the characteristic of natural music; has often found, when he has been wandering wildly through the mountains of Wales, and has sung away without thinking *what* he sang, that he has invariably detected himself omitting the seventh and fourth." The following entry is also interesting, at p. 341: "Dined with Power, to meet Bishop ——. He mentioned a good story to prove how a musician's ear requires the extreme seventh to be resolved. Sebastian Bach, one morning getting out of bed for some purpose, ran his fingers over the keys of the piano as he passed, but when he returned to bed found he could not sleep. . . . . . At length he recollected that the last chord he struck was that of the seventh; he got up again, resolved it, and then went to bed and slept as comfortably as he could desire."

The preceding series of arguments and illustrations have brought the subject to the point from whence it was commenced—namely, the unconscious or reflex action of the cerebrum. Perhaps enough has been stated to establish these two prime truths,—1. That the unconsciously working principle of intelligence manifested in the construction and instincts of vegetables and of animals, is identical with the unconsciously working principle of intelligence manifested in the construction and functions of the human cerebrum; 2. That the human mind is none other than this unconscious principle of intelligence individualized, become cognizant thereby of its own workings in the cerebrum, and deriving its ideas from its own constructive or material changes in the organ of mind. To demonstrate more clearly the unity of origin and action of the two forms of intelligence, and the application of the doctrine to practical uses, I will now add some further illustrations, taking the intellectual instincts as a starting-point.

The appreciation of the Beautiful, in connexion with the pleasures of sense, is a familiar fact to the moralist and the philosopher. In man it is first felt rightly with the complete evolution of the system, and when he is become capable of reproducing the species: just as, at the same stage of evolution, his beauty is most perfect. No idea of the unconscious principle of intelligence is more universal than this. In many of the phanerogamous plants the period of formation of the primordial cell (or union of sperm-cell and germ-cell—fertilization of the ovum) is marked by a display of grace of form and beauty of colour in the appendages to the sexual organs, which it is man's highest ambition to rival successfully. These appendages are formed out of what are the analogues of the male organs. In the insect-world, the brief period of fertilization is also the period of perfect development; in some of these, as the *Lepidoptera*, there is a gorgeous decoration of the animal, and more particularly in the male. In fishes, birds, and mammals, puberty is also characterized by the development of ornaments more or less striking, but more especially on the male; scales brightly coloured, gorgeous feathers (as in humming-birds, and the Gallinaceæ), and horns, manes, beards, are of this kind. In the human female, the hair, the mammæ, and the subcutaneous fat, are undoubtedly analogous structures. The conscious mind displays in man a similar law of action; the gay attire of the lover, and the glories of bridal dress and decoration, are but evolutions of the same great idea of the unconscious mind.

While thus in creation the outward form is æsthetically a unity, so also are minor sources of sensation. Many animals are attracted by *scents* developed during reproductive activity—insects, fishes, and mammals, not excepting man; it is during this period that flowering plants give off their sweets. *Sounds*, of a more or less musical character, are emitted by insects, birds, and mammals, during the same period—perhaps almost exclusively by the males; in this respect the analogy (as to plants and the lower animal forms) is defective. In man, the taste for poetry, music, sculpture, and the decorative arts, is only fully developed with the evolution of the reproductive organs, while it is exalted as to one of these by their special activity. The ballad "to his mistress' eye-brow" of the lover, is the exact analogue of the song of the cicada, or of the male song-bird.

I have given a practical application to these views in an attempt to investigate the nature and origin of hysterical affections, and to this work I would refer the reader.* If the instincts of man, and vegetables, and animals, be collated in reference to the continuance of the species, they will be found to be inseparably connected with every kind of both æsthetic and constructive art, in every form of organism.

Ordinary dreaming, somnambulism, clairvoyance, delirium of every sort, in-

* "A Treatise on the Nervous Diseases of Women." Longmans and Co.

sanity, and other forms of disordered cerebral action, are important changes in the states of the consciousness in reference to the representative faculty. There can be little doubt that these changes have their correlative changes in the vesicular neurine itself, although the demonstration is not easy. In the action of alcohol, chloroform, opium, Indian hemp, &c., on the blood, and, through the blood, on the cerebral tissue, we have, however, an undeniable proof that there are instances in which these changes in the consciousness *do* depend upon changes in the cells of the vesicular neurine, for the invariable connexion of antecedent and consequent is most clearly made out in reference to these. It is a doctrine generally entertained, that narcotic poisons have each a special action upon special portions of the encephalon; but I think there is considerable doubt to what extent, at least, this should be admitted. The difference may be rather in the mode of action than the locality selected; for it by no means follows that these poisons must necessarily affect the vesicular neurine so as to alter the states of the consciousness. On the contrary, it is exceedingly probable (if the proposition I have advanced be granted—namely, that the function of the nerve-cells is only the result of a specialization and evolution of a more general function inherent in all cells), that the latter participate with the former in the changes which the so-called narcotic poisons induce. That this is so with some of them is undeniable, and I will proceed to show this with reference to opium, hoping at the same time to demonstrate the *principles* (in opposition to our empirical knowledge) by which the administration of the drug should be regulated.

The first result of the action of opium on the tissues is to exalt the feeling of corporeal well-being; it is, therefore, congruous with the normal action of those tissues. Its power of actually sustaining the vital powers is well illustrated by the use made of it by messengers and others in the East, both for themselves and their horses, when they have to undergo prolonged labour with little sustenance. Acting upon the organs of self-consciousness and thought, it again exalts the feeling of pleasure in connexion with their action and the states of consciousness arising therefrom. To the wounded spirit it is described, by one who tried it largely, "as an assuaging balm;" and as building out of the fantastic imagery of the brain, "cities and temples beyond the art of Phidias or Praxiteles—beyond the splendour of Babylon and Hekatompylos." Now this being the action of opium upon tissues wherein consciousness plays, we may infer that it has an analogous action on tissues apart therefrom; and this experience shows to be the case, for there is perhaps no remedy which more facilitates a return to normal action in those tissues when the seat of sloughing wounds, or when the vital reaction is below *par*, than opium. So, also, when the nutrition or vital action of the vesicular neurine is imperfect from like causes, as in asthenic neuralgia, the various forms of melancholia (especially those connected with excessive use or action of the organ), and the asthenic forms of delirium and delirious mania, opium is the most certain medicinal agent. Those who have studied these varied uses of opium empirically, will recognise the justice of these statements as to its widely-different therapeutic applications, and will readily understand that the common link which binds them together in one therapeutic category, is the unity of function of cells in relation to the predetermined arrangements of the unconscious mind. The irritability of a chronically inflamed mucous surface, and the irritability of a nerve or sensorial centre, are not essentially different pathologically; on each, opium acts medicinally in a way also not essentially different. I would call special attention to this point in my system, as one of exceeding value in therapeutics, for if that system be well-founded, we can interpret the so-called vital phenomena by those which involve consciousness, and *vice versâ;* for the latter being nothing more than the workings of the unconscious soul reaching the consciousness through a special apparatus evolved for the purpose, and the works of the unconscious soul not reaching the consciousness, being vital phenomena, the one can be substituted for the other in our inquiries, so far as the bio-chemical changes in the tissues are involved.

I had intended to have illustrated the nature of the Will (a state of self-consciousness) by an application of these views to the phenomena of motion in organisms, whether animal or vegetable; this must form a subject for further and separate inquiry. As to the doctrines advanced, I may be permitted to say, that they really constitute only a small portion of a general system of mental philosophy, and are therefore of necessity presented in a fragmentary shape. In thus opening out a new and altogether uninvestigated series of related phenomena, I think it right to make some remarks which may be of use in explaining my views and guiding the thinker and observer.

I have constantly made use of the term unconscious principle of intelligence or *mind*. By that term I mean simply to designate that principle of intelligence which is manifested in *all* the phenomena of the universe, so far as they are known, and whether cosmic or organic, in virtue of which all things tend to Good. It is a principle, according to my views, as universally operative, as devoid of personality, and as certain and definite in its laws of action as the force of gravity, and is the primary and essential element of the conscious mind. I term it the *unconscious* mind because to us it so appears to be in its operation in organisms; for although there can be no doubt whatever that it proceeds from the great creative Intelligence, yet the laws of the inductive philosophy forbid us to investigate its relations to the Deity, since these are clearly beyond the reach of philosophical observation and experiment. Like the force of gravity, it is a property of matter, and like it, probably dependent upon an immediate volition of the Deity. Speculations as to its nature and relations have been current in every age, and need not be multiplied now. It has been conceived to be God himself; a doctrine which has constituted the foundation of Pantheistic and analogous systems of theology; or under the term Nature, it has occupied the place of the Deity in Atheistic systems; or in Deistic systems, has been viewed as a special moral agent. In Cosmogony, it has been considered as a *hylozoic* principle animating the world, as if the latter were an animal; or, in relation to natural history and physiology, has been considered as the *anima*, plastic nature (Cudworth), the Archæus, the vital principle, the *vis nervosa*, &c. All these speculations I wish to avoid, preferring to investigate its laws of action through its phenomena: these are two-fold: the changes it operates in matter, in reference to the ends it has in view, as manifested by phenomena; and the changes in the states of the consciousness, consequent on those material changes. When these laws have been determined and settled, in part at least, we shall be in a position to determine more satisfactorily than hitherto, the relations of the self-conscious mind to organization, the nature of Truth, and the limits of moral responsibility; or, in other words, to establish psychology, metaphysics, and moral philosophy on a more definite basis.

I have repeatedly used the term psychical *substrata*. By this I do not mean to imply a certain material arrangement of cells or their elements only, but such an arrangement that a fixed order of successional changes, or plan of action, may be impressed upon them. Thus each primordial or embryonic cell has its psychical substrata, in virtue of which there is a continuous series of successional changes in a fixed, predetermined order, and according to a fixed plan. So, also, in those cell-masses (or vesicular neurine) appropriate to special ideas, there are psychical substrata, in virtue of which there is a constant construction of new cells, corresponding to those new states of the consciousness comprised under the general term *development of ideas*, the ideas being developed and the new cells constructed according to a fixed and predetermined law of development. The substrata have *potential* properties—that is to say, they contain the germs of further and indefinite series of future changes, as well as properties in actual use in relation to the external world.

## Figures and Tables

**Figure f1:**